# Quality of Multicenter Studies Using MRI Radiomics for Diagnosing Clinically Significant Prostate Cancer: A Systematic Review

**DOI:** 10.3390/life12070946

**Published:** 2022-06-23

**Authors:** Jeroen Bleker, Thomas C. Kwee, Derya Yakar

**Affiliations:** Medical Imaging Center, Departments of Radiology, Nuclear Medicine and Molecular Imaging, University Medical Center Groningen, University of Groningen, Hanzeplein 1, 9700 RB Groningen, The Netherlands; t.c.kwee@umcg.nl (T.C.K.); d.yakar@umcg.nl (D.Y.)

**Keywords:** radiomics, multicenter MRI, prostate cancer

## Abstract

*Background:* Reproducibility and generalization are major challenges for clinically significant prostate cancer modeling using MRI radiomics. Multicenter data seem indispensable to deal with these challenges, but the quality of such studies is currently unknown. The aim of this study was to systematically review the quality of multicenter studies on MRI radiomics for diagnosing clinically significant PCa. *Methods:* This systematic review followed the 2020 Preferred Reporting Items for Systematic Reviews and Meta-Analyses (PRISMA) checklist. Multicenter studies investigating the value of MRI radiomics for the diagnosis of clinically significant prostate cancer were included. Quality was assessed using the checklist for artificial intelligence in medical imaging (CLAIM) and the radiomics quality score (RQS). CLAIM consisted of 42 equally important items referencing different elements of good practice AI in medical imaging. RQS consisted of 36 points awarded over 16 items related to good practice radiomics. Final CLAIM and RQS scores were percentage-based, allowing for a total quality score consisting of the average of CLAIM and RQS. *Results:* Four studies were included. The average total CLAIM score was 74.6% and the average RQS was 52.8%. The corresponding average total quality score (CLAIM + RQS) was 63.7%. *Conclusions:* A very small number of multicenter radiomics PCa classification studies have been performed with the existing studies being of bad or average quality. Good multicenter studies might increase by encouraging preferably prospective data sharing and paying extra care to documentation in regards to reproducibility and clinical utility.

## 1. Introduction

Prostate cancer (PCa) has a high incidence rate and causes a high absolute number of deaths [1]. Improvements in PCa diagnosis have been made with the introduction of MRI and the prostate imaging and reporting data system (PI-RADS) [2,3,4]. Computer aided detection (CAD) techniques [5] have shown promise for additional diagnostic improvements of MRI. Particularly, many efforts on improvement of MRI-based PCa diagnosis were based on some form of machine learning [6]. Nevertheless, the correct diagnosis of clinically significant (CS) PCa (i.e., PCa pathologically defined as International Society of Urological Pathology grade ≥ 2), remains difficult, even when including novel strategies such as miRNA [7]. With the introduction of radiomics [8], a seemingly simple technique was brought forward as a possible valuable addition to machine learning models for CS PCa. Radiomics extracts image information normally invisible to the human eye, which can be used to quantify tumor phenotypes [8]. Over the years, the combination of radiomics and machine learning has shown its strengths in MRI-based CS PCa diagnosis [9].

However, models based on radiomics data are not without weaknesses [10,11,12]. While building a working radiomics model is a relatively straightforward process, attaining a generalizable radiomics model that outperforms a radiologist is considerably more complex. Multiple guidelines have been suggested to help with development and improve both machine learning models and radiomics in healthcare [13,14,15]. Mongan et al. recently introduced the checklist for artificial intelligence in medical imaging (CLAIM). CLAIM can be used as a guideline for authors and reviewers of artificial intelligence papers in healthcare [14]. In an effort to tackle the lack of radiomics standardization, Zwanenburg et al. compiled the extensive image biomarker standardization initiative [13]. Furthermore, Lambin et al. introduced the radiomics quality score (RQS) to fill the need for homogeneous radiomics evaluation criteria and reporting guidelines [15].

Multicenter large scale data appear invaluable to create generalizable radiomics models that are clinically useful [10,16], and that may assist or outperform radiologists in diagnosing CS PCa on MRI [17]. Herein lies the issue with current radiomics PCa studies and reviews, with almost all of them having been developed on a single center dataset or focus on single center performance [9,18]. Good single center performance does not guarantee good multicenter performance, while the opposite does seem to be true [10,16]. To our knowledge, no previous review has focused specifically on multicenter radiomics. Therefore, this study aimed to systematically review the quality of multicenter radiomics studies for the diagnosis of CS PCa according to CLAIM and RQS.

## 2. Materials and Methods

This systematic review followed the 2020 Preferred Reporting Items for Systematic Reviews and Meta-Analyses (PRISMA) checklist.

### 2.1. Eligibility Criteria

Studies were potentially eligible for inclusion if their goal was to investigate the diagnosis of clinically significant PCa using multicenter radiomics MRI data. Studies were included for total study screening if the following terms could be found in either the title, abstract, or key words: radiomics, prostate cancer, MRI, and multicenter. Term synonyms, abbreviations, and their closest fitting medical subject heading (MeSH) terms were included in the search strategy. Only original research was eligible for inclusion. Exclusion criteria were: studies with less than 150 patients (less than 150 patients appears insufficient for learning the patterns in usually complex multicenter data [10,19]), and studies exclusively focusing on the diagnosis of extraprostatic tumor extension and PCa recurrence.

### 2.2. Search Strategy and Sources

Scopus, Embase, Web of Science, and Pubmed were searched in February 2022. The following search string was used: ((radiomics) OR (feature-based) OR (feature based)) AND ((prostate) OR (PCA) OR (PC) OR (prostate cancer)) AND ((MRI) OR (bpMRI) OR (magnetic resonance imaging)) AND ((multicenter) OR (multi-center) OR (collaborative) OR (multi-institutional)). Only English search terms were used.

### 2.3. Study Selection and Data Extraction

Reference files extracted from the searched databases were added to Mendeley (Version 1.19.8, Elsevier, London, UK). Duplicate papers were removed by the internal duplicate scanner. A single reviewer (JB, with 4 years of hands-on research experience in MRI radiomics of PCa) checked each title, abstract, and key terms manually for their fit to the specified inclusion and exclusion criteria. All remaining eligible studies were read by the same reviewer and graded according to CLAIM and RQS [14,15]. Grades were checked for bias by another reviewer (CR, with 1.5 years of hands-on research experience in MRI research). The number of included patients and/or lesions, number of institutions, use of multicenter data, and available model performance metrics (area under the curve, sensitivity, specificity, etc.) were extracted.

### 2.4. CLAIM

CLAIM consists of 42 quality items, with special focus on ground truth, data partitions, modeling, training, evaluation, and performance. CLAIM was divided into six sections/topics starting with title/abstract, then introduction, Methods, Results, Discussion, and finally other information which was the last main section for the CLAIM checklist. Each of the sections, subsections, and their 42 items with a short description can be found in Table 1.

Argumentation for the inclusion of each of the 42 items can be found in the original publication by Mongan et al. [14]. Some of the CLAIM items were not applicable to some of the included studies. For example, “tools used for annotation” requires manual annotation by multiple experts. If a study used an automatic annotation or no annotation at all, this CLAIM item was scored as not applicable. Each CLAIM checklist item was seen as equally important and worth 1 point with a maximum of 42 points.

### 2.5. RQS

The RQS consists of 16 detailed quality items mainly focusing on reproducibility and validation of radiomics. The first four RQS items were mostly scanner and protocol related: the detailed documentation/description of the image protocol, the requirement for multiple segmentations, phantom studies on all scanners, and imaging at multiple time points. The next three RQS items focused on everything feature related: setup of feature reduction or adjustment for multiple testing, multivariable analysis with non-radiomics features, and detection and discussion of biological feature correlates. The following three RQS items contained statistic quality checks: determination of risk groups through cut-off analysis, inclusion of discrimination statistics, and reporting the calibration statistics. The eleventh RQS item was standalone, seen as extra important, and checked the study for prospective trial database registry. The eleventh RQS item was followed by two performance-related RQS items: detailed execution of validation, and comparison to gold standard. The last three RQS items were more general with one checking the potential clinical utility, the second checking for the inclusion of a cost-effectiveness analysis, and finally if the study was open source. There was minimal overlap with the 42 CLAIM items. Some items of the RQS checklist are seen as more important than others and are assigned more points when fulfilled. RQS item argumentation can be found in Lambin et al. [15]. A Tabular overview of each detailed RQS item and its weight can be found in Appendix A.

### 2.6. Data Analysis

Each of the CLAIM items scored as not applicable was deducted from the total of 42 CLAIM items before calculating the percentage of CLAIM items that was fulfilled by each study. RQS percentage scores were calculated based on the RQS points table and their maximum of 36. Excellent studies should be able to achieve 85–90% for both CLAIM and RQS, a percentage estimated based on the CLAIM and RQS review by our clinical and technical radiomics PCa experts (9 years and 4 years experience). Averages and standard deviations of the total CLAIM and RQS were calculated.

## 3. Results

**Figure 1:**
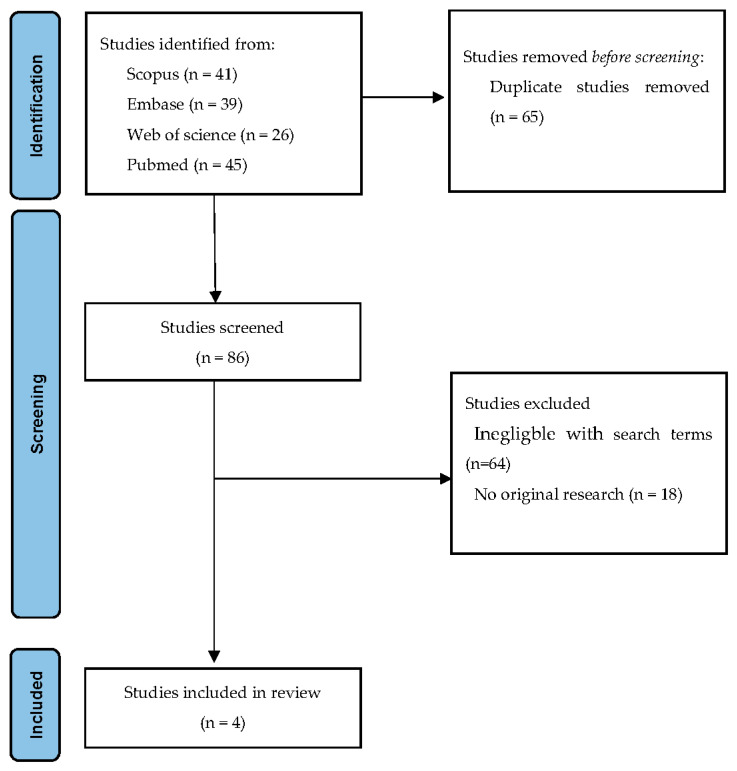
PRISMA 2020 Flow diagram.

The search strategy resulted in 151 results, 41 from Scopus, 39 from Embase, 26 from Web of science, and 45 from Pubmed. A total of 65 duplicate studies were removed. Furthermore, 86 studies remained for screening their title, abstract, and key terms according to the inclusion and exclusion criteria. In addition, 82 studies were removed because they were clearly ineligible or because it did not concern original data. Four studies remained and were included in this review. The corresponding PRISMA flow diagram can be found in Figure 1.

### 3.1. Description of Included Studies

The first study by Bleker et al. [10] aimed to investigate a previously developed radiomics-based biparametric MRI (T2-weighted imaging, diffusion-weighted imaging) approach for the diagnosis of clinically significant peripheral zone PCa. Their study used both a single center, single vendor dataset and a multicenter, multivendor dataset for validation and model development. The study population consisted of 262 single center lesions from a single institution and vendor and another set of 262 multicenter lesions originating from nine different institutions and two vendors. Both sets were split into 171 training lesions and 91 test lesions. The radiomics model developed on single center data showed a performance reduction of 27% when validated on multicenter data (AUC 0.82 vs. 0.59). A multicenter developed model achieved a multicenter validation AUC of 0.75 and a single center validation of 0.66.

The second study by Castillo et al. [20] aimed to compare the performance of a multiparametric MRI radiomics model with that of a deep learning model for the diagnosis of clinically significant PCa. Their study included 271 patients from one institution and three external sets of 195, 100, and 78 patients from three other institutions. The external datasets were used as test datasets. Their radiomics model achieved AUCs of 0.88, 0.91, and 0.65, while the deep learning model achieved AUCs of 0.70, 0.73, and 0.44 on the three test sets, respectively.

The third study by Lim et al. [21] aimed to develop and evaluate both a T2-weighted-based radiomics model and apparent diffusion coefficient (ADC)-based radiomics model to diagnose clinically significant PCa in PI-RADS category 3 lesions. Their study population consisted of 158 patients with 160 PI-RADS category 3 lesions from two different institutions. The T2-weighted radiomics model achieved an AUC of 0.547 and the ADC-based model, an AUC of 0.684

The fourth study by Montoya Perez et al. [22] aimed to develop and validate biparametric MRI radiomics and blood kallikrein (peptidase family of which prostate specific antigen (PSA) is a member) models for the detection of clinically significant PCa. Their study population consisted of 543 patients from four different institutions. The total study population was divided equally in data split 1 and data split 2, which were both split into train, validation, and test sets. AUCs for the biparametric MRI radiomics model were 0.83 and 0.83 for both test sets, which were not significantly different from a prediction made using a risk stratification scheme such as PI-RADSv2 [4].

### 3.2. Quality of Included Studies

CLAIM evaluation for each of the studies included in this review can be found in Table 2 and the RQS evaluation in Table 3.

CLAIM scores ranged from 71.1% to 80.6% and RQS ranged from 44.4% to 58.3%. Study design, ground truth labeling, data partitioning, and model training scored particularly well. While items related to data preprocessing, de-identification, and clinical use scored terribly. For radiomics, the feature reduction, model performance, and proper validation with correct ground truth showed excellent results. More specific items related to feature reproducibility, prospective data, and cost-effectiveness analysis are generally lacking.

## 4. Discussion

This systematic review investigated the quality of currently available multicenter MRI radiomics studies for the diagnosis of clinically significant PCa, with quality defined according to CLAIM [14] and RQS [15]. The first important finding of our systematic review is that the number of multicenter radiomics PCa classification studies eligible is low. Most literature seems to agree that large scale multicenter datasets are required for radiomics [19,23,24]. Lagging use of multicenter datasets for radiomics diagnosis of clinically significant PCa has been observed to be related to technical challenges, patient privacy, and data security issues [25]. Developments for faster and secure data sharing and storage, and data partnerships between hospitals and corporations, may be able to circumvent these challenges [25,26]. Overall, data sharing initiatives are occurring more frequently [27] and publicly available datasets are increasing [28]. This will hopefully increase the number of multicenter studies in the field of MRI radiomics of PCa. The second important finding of this systematic review is that the few studies which did use multicenter PCa data scored reasonably well on CLAIM (74.6%) and worse on RQS (52.8%). Items related to experiment setup and model training and validation performed generally very well. While items related to data preprocessing, data de-identification, feature reproducibility, and implications for practice did not.

Interestingly, models developed using multicenter radiomics data seem to struggle with diagnostic performance. Both Lim et al. [21] and Bleker et al. [10] developed models that are generalizable (i.e., maintain diagnostic performance in external datasets), but they did not achieve AUC scores that are higher than 0.75. Both Bleker and Lim et al. believe this lower performance is related to multicenter data heterogeneity (differences in included sequences and image intensity related to vendor, scanner, and protocol variability) and data processing, which does not fully combat this heterogeneity. Recent literature confirms this observation [13,23,24,29] and more studies should be performed on multicenter data processing. Another interesting addition to multicenter PCa radiomics might be the inclusion of clinical features. Montoya Perez et al. showed that the introduction of clinical features to their PCa radiomics model showed potential and stability in the 10-fold cross validation of the multicenter test dataset. [22]. Yet another improvement might be a more diverse approach to model development where instead of one single model, multiple models are developed and combined [30]. Castillo et al. [20] developed a combined model (consisting of 100 different models) which outperformed its cross validated training score on an external validation dataset (*n* = 195, ProstateX [31], General Electric vs. Siemens AUC 0.83 vs. 0.91). However, this same combined model showed a reduction in performance on another external dataset from a different vendor (*n* = 78, General electric vs. Philips, AUC 0.83 vs. 0.65). Due to the mismatch in generalization, more research on combined models for multicenter radiomics PCa classification is recommended.

Besides challenges in data sharing and multicenter performance, quite a few general model quality deductions could be made. According to the CLAIM scores in Table 1, authors could benefit from including more details in their studies. Documentation of settings related to MR protocols, anonymization, and radiomics feature extraction that directly influence reproducibility is lacking. This documentation is especially important for radiomics since a major challenge is reproducibility [12,32]. A recent review by Midiri et al. also confirmed that reproducibility and standardization remain main challenges for radiomics [33]. CLAIM item 9 related to the total description of data preprocessing is the first item where all authors lacked any scores caused by missing details. All settings (i.e., voxel spacings, algorithms used, scaling, etc.) need to be included to make processing reproducible. Further issues were: handling of missing data, missing anonymization, removal of outliers, and detailed specification of any software used was missing completely. CLAIM item 12 related to the description of the data anonymization protocol was the second item all authors failed. No information on any of the anonymization approaches was included. Finally, the description of future clinical implementation tested by CLAIM item 39 was also failed by all authors. For the RQS, it was observed that the prospective study design, “Phantom studies on all scanners” and “Imaging at multiple time points—Delta radiomics” was likewise lacking. While prospective study design is a critical shortcoming [33], phantom use and Delta radiomics might be less important.

This systematic review had some limitations. First, there were slight differences in study goals, patient numbers, labels, and datasets among the four included studies, which makes direct quality comparison slightly more difficult. Nevertheless, some comparison is warranted since a certain degree of generalization is expected with multicenter data and trends in quality issues can be deducted. Second, the RQS study was published back in 2017 [15]. Radiomics and artificial intelligence is a rapidly developing field which makes the current RQS slightly outdated. Third, CLAIM is focused on deep learning models and not feature-based radiomics models, which resulted in various non-applicable checklist items for each included study.

In conclusion, a very small number of multicenter radiomics PCa classification studies have been performed with the existing studies being of bad or average quality. Good multicenter studies might increase by encouraging preferably prospective data sharing and paying extra care to documentation in regards to reproducibility and clinical utility.

## Figures and Tables

**Table 1:** CLAIM checklist table with three columns containing the section/subsection, CLAIM item number, and the description of the item.

Title/Abstract		
	1	Identification as a study of AI methodology, specifying the category of technology used (e.g., deep learning)
	2	Structured summary of study design, methods, results, and conclusions
**Introduction**		
	3	Scientific and clinical background, including the intended use and clinical role of the AI approach
	4	Study objectives and hypotheses
**Methods**		
Study Design	5	Prospective or retrospective study
	6	Study goal, such as model creation, exploratory study, feasibility study, non-inferiority trial
Data	7	Data sources
	8	Eligibility criteria: how, where, and when potentially eligible participants or studies were identified (e.g., symptoms, results from previous tests, inclusion in registry, patient-care setting, location, dates)
	9	Data pre-processing steps
	10	Selection of data subsets, if applicable
	11	Definitions of data elements, with references to Common Data Elements
	12	De-identification methods
	13	How missing data were handled
Ground Truth	14	Definition of ground truth reference standard, in sufficient detail to allow replication
	15	Rationale for choosing the reference standard (if alternatives exist)
	16	Source of ground-truth annotations; qualifications and preparation of annotators
	17	Annotation tools
	18	Measurement of inter- and intrarater variability; methods to mitigate variability and/or resolve discrepancies
Data Partitions	19	Intended sample size and how it was determined
	20	How data were assigned to partitions; specify proportions
	21	Level at which partitions are disjoint (e.g., image, study, patient, institution)
Model	22	Detailed description of model, including inputs, outputs, all intermediate layers and connections
	23	Software libraries, frameworks, and packages
	24	Initialization of model parameters (e.g., randomization, transfer learning)
Training	25	Details of training approach, including data augmentation, hyperparameters, number of models trained
	26	Method of selecting the final model
	27	Ensembling techniques, if applicable
Evaluation	28	Metrics of model performance
	29	Statistical measures of significance and uncertainty (e.g., confidence intervals)
	30	Robustness or sensitivity analysis
	31	Methods for explainability or interpretability (e.g., saliency maps), and how they were validated
	32	Validation or testing on external data
**Results**		
Data	33	Flow of participants or cases, using a diagram to indicate inclusion and exclusion
	34	Demographic and clinical characteristics of cases in each partition
Model performance	35	Performance metrics for optimal model(s) on all data partitions
	36	Estimates of diagnostic accuracy and their precision (such as 95% confidence intervals)
	37	Failure analysis of incorrectly classified cases
**Discussion**		
	38	Study limitations, including potential bias, statistical uncertainty, and generalizability
	39	Implications for practice, including the intended use and/or clinical role
**Other information**		
	40	Registration number and name of registry
	41	Where the full study protocol can be accessed
	42	Sources of funding and other support; role of funders

**Table 2:** Checklist for artificial intelligence in medical imaging evaluation for each of the five studies included in the review. If the study fit the total CLAIM item description, a score of 1 was awarded. For example, item 1: “Indicate the use of the AI techniques—such as “deep learning” or “random forests”—in the article’s title and/or abstract” requires detailed mention of all AI techniques used. If one or more is missing, a zero was given. N/A stands for non-applicable and is used when the specific item does not fit with the goal or approach of the study. Each N/A reduces the possible total score (42—number of N/As) that is used for calculating the percentage of items fulfilled.

Domain	Item	Bleker et al. [10]	Castillo et al. [20]	Lim et al. [21]	Montoya Perez et al. [22]
**Title/Abstract**					
	1	0	0	1	0
	2	1	1	1	1
**Introduction**					
	3	1	1	1	1
	4	1	0	0	0
**Methods**					
Study Design	5	1	1	1	1
	6	1	1	1	1
Data	7	1	1	1	1
	8	1	1	1	0
	9	0	0	0	0
	10	N/A	N/A	N/A	N/A
	11	1	1	1	1
	12	0	0	0	0
	13	0	1	0	1
Ground Truth	14	1	1	1	1
	15	1	1	1	1
	16	1	0	1	1
	17	N/A	0	1	1
	18	N/A	N/A	0	N/A
Data Partitions	19	1	1	1	1
	20	1	1	0	1
	21	1	1	1	1
Model	22	1	1	1	1
	23	0	1	1	0
	24	1	1	1	1
Training	25	1	1	1	1
	26	1	1	1	1
	27	N/A	1	N/A	N/A
Evaluation	28	1	1	1	1
	29	1	0	1	1
	30	1	1	1	0
	31	1	1	0	1
	32	1	1	1	1
**Results**					
Data	33	1	1	1	1
	34	0	0	1	1
Model performance	35	1	1	0	1
	36	1	1	1	1
	37	1	0	0	0
**Discussion**					
	38	1	1	1	1
	39	0	0	0	0
**Other information**					
	40	N/A	N/A	N/A	N/A
	41	N/A	N/A	N/A	N/A
	42	1	0	1	1
Total score percentage		80.6 (29/36)	71.1 (27/38)	71.1 (27/38)	75.7(28/37)

RQS grading for each of the studies included in this review can be found in Table 2.

**Table 3:** Radiomics quality scores and total percentages for each of the studies included in this review. Total maximum score that could be achieved is 36 points.

RQS	Bleker et al. [10]	Castillo et al. [20]	Lim et al. [21]	Montoya Perez et al. [22]
Image Protocol Quality	2	2	2	1
Multiple segmentations	1	1	1	0
Phantom Study on all scanners	0	0	0	0
Imaging at multiple time points	0	0	0	0
Feature reduction or adjustment feature reduction or adjustment for multiple testing	3	3	3	3
Multivariable analysis with non radiomics features	0	0	0	1
Detect and discuss biological correlates	0	0	0	1
Cut-off analyses	0	0	0	0
Discrimination statistics	2	2	2	2
Calibration statistics	1	1	1	1
Prospective study registered in a trial database	0	0	0	0
Validation	5	5	3	3
Comparison to ‘gold standard’	2	2	2	2
Potential clinical utility	2	2	2	2
Cost-effectiveness analysis	0	0	0	0
Open science and data	2	3	0	3
Total score percentage	55.6 (20/36)	58.3 (21/36)	44.4 (16/36)	52.8 (19/36)

## Data Availability

Protocol and data and analysis used are available on request.

## Appendix A

**Table A1:** The radiomics quality score: RQS.

Criteria		Points
1	Image protocol quality—well-documented image protocols (for example, contrast, slice-thickness, energy, etc.) and/or usage of public image protocols allow reproducibility/replicability	+1 (if protocols are well-documented) +1 (if public protocol is used)
2	Multiple segmentations—possible actions are: segmentation by different physicians/algorithms/software, perturbing segmentations by (random) noise, segmentation at different breathing cycles. Analyze feature robustness to segmentation variabilities	+1
3	Phantom study on all scanners—detect inter-scanner differences and vendor -dependent features. Analyze feature robustness to these sources of variability	+1
4	Imaging at multiple time points—collect images of individuals at additional time points. Analyze feature robustness to temporal variabilities (for example, organ movement, organ expansion/shrinkage)	+1
5	Feature reduction or adjustment for multiple testing—decreases the risk of overfitting. Overfitting is inevitable if the number of features exceeds the number of samples. Consider feature robustness when selecting features	−3 (if neither measure is implemented) +3 (if either measure is implemented)
6	Multivariable analysis with non radiomics features (for example, EGFR mutation)—is expected to provide a more holistic model. Permits correlating/inferencing between radiomics and non radiomics features	+1
7	Detect and discuss biological correlates—demonstration of phenotypic differences (possibly associated with underlying gene-protein expression patterns) deepens understanding of radiomics and biology	+1
8	Cut-off analyses—determine risk groups by either the median, a previously published cut-off or report a continuous risk variable. Reduces the risk of reporting overly optimistic results.	+1
9	Discrimination statistics—report discrimination statistics (for example, C-statistic, ROC curve, AUC) and their statistical significance (for example, p-values, confidence intervals). One can also apply resampling methods (for example, bootstrapping, cross validation)	+1 (is a discrimination statistic and its statistical significance are reported) +1 (if a resampling method technique is also applied)
10	Calibration statistics—report calibration statistics (for example, Calibration-in-the-large/slope, calibration plots) and their statistical significance (for example, p-values, confidence intervals). One can also apply resampling methods (for example, bootstrapping, cross validation)	+1 (is a calibration statistic and its statistical significance are reported) +1 (if a resampling method technique is also applied)
11	Prospective study registered in a trial database— provides the highest level of evidence supporting the clinical validity and usefulness of the radiomics biomarker	+7 (for prospective validation of a radiomics signature in an appropriate trial)
12	Validation—the validation is performed without retraining and without adaption of the cut-off value, provides crucial information with regard to credible clinical performance	−5 (if validation is missing) + 2 (if validation is based on a dataset from the same institute) +3 (if validation if based on a dataset from another institute) +4 (if validation is based on two datasets from two distinct institutes) +4 (if the study validates a previously published signature) +5 (if validation is based on three or more datasets from distinct institutes) Datasets should be of comparable size and should have at least 10 events per model feature
13	Comparison to ’gold standard’—assess the extent to which the model agrees with/is superior to the current ’gold standard’ method (for example, TNM-staging for survival prediction). This comparison shows the added value of radiomics	+2
14	Potential clinical utility—report on the current and potential application of the model in a clinical setting (for example decision curve analysis)	+2
15	Cost-effectiveness analysis—report on the cost-effectiveness of the clinical application (for example, QALYs generated)	+1
16	Open science and data—make code and data publicly available. Open science facilitates knowledge transfer and reproducibility of the study	+1 (if scans are open source) +1 (if region of interest segmentations are open source) +1 (if code is open source) +1 (if radiomics features are calculated on a set of representative ROIs and the calculated features and representative ROIs are open source)
		Total points (36 = 100%)
